# A randomized, double-blinded, placebo-controlled, crossover study of the HCN channel blocker ivabradine in a capsaicin-induced pain model in healthy volunteers

**DOI:** 10.1038/s41598-022-22309-7

**Published:** 2022-10-14

**Authors:** Satoshi Tanaka, Takashi Ishida, Kumiko Ishida, Satoshi Fuseya, Mariko Ito, Akiyuki Sakamoto, Mikito Kawamata

**Affiliations:** Department of Anesthesiology and Resuscitology, Shinshu University School of Medicine, Matsumoto, Nagano 390-8621 Japan

**Keywords:** Chronic pain, Pain management

## Abstract

Hyperpolarization-activated cyclic nucleotide-gated (HCN) channels have been focused on as a potential therapeutic target for inflammatory and neuropathic pain in rodent models. However, roles of HCN channels in human pain states have been scarcely investigated. We evaluated analgesic effects of 2-day administration of ivabradine, the only clinically available HCN channel blocker, on a capsaicin pain model in a randomized, double-blinded, placebo-controlled, crossover study. Twenty healthy adult subjects (18 males, 2 females) received ivabradine (5–7.5 mg) or a placebo 3 times in 2 days. Then capsaicin (0.5%) was topically applied on the volar forearm for 30 min. The primary outcome was capsaicin-induced spontaneous pain. The secondary outcomes included heat-pain threshold (HPT), flare size, and areas of secondary punctate mechanical hyperalgesia (PMH) and secondary dynamic mechanical allodynia (DMA). There was no significant difference in spontaneous pain (*p* = 0.7479), HPT (*p* = 0.7501), area of PMH (*p* = 0.1052) or flare size (*p* = 0.5650) at 30 min after capsaicin application between the groups. In contrast, the area of DMA in the ivabradine group was significantly smaller (*p* < 0.001) than that in the placebo group. HCN channels may be differentially involved in the various pain signal transmission pathways in humans.

## Introduction

Recently, hyperpolarization-activated cyclic nucleotide-gated (HCN) channels have been focused on as a potential therapeutic target for inflammatory and neuropathic pain^1,2^. HCN channels are activated by membrane hyperpolarization and play an important role in controlling and facilitating neuronal excitability^3^. In mammals, HCN channels consist of four isoforms (HCN1–4), which are responsible for the transport of sodium and potassium ions. HCN1 and HCN2 are expressed in the dorsal root ganglion (DRG), spinal cord, and some brain regions^1,2^ and are thought to be involved in pain transmission. HCN3 and HCN4 are rarely expressed in DRG neurons^4^, while HCN4 isoform is the major component of pacemaker channels in the sinoatrial node^3^.

Ivabradine, which inhibits all of the four HCN channels with similar IC_50_ values^5^, has been approved for clinical use in the treatment of angina pectoris and heart failure across Europe and the United States^6–8^. Ivabradine can reduce the resting heart rate mainly through blockade of HCN4 channels with few serious side effects^3,5^. In addition, it has been reported that ivabradine alleviates neuropathic pain^9–11^ and inflammatory pain^10,12^ in rodent models. Ivabradine reduced pain behavior in the second phase (inflammatory) in a formalin model but had no effect in the early nociceptive phase^10^. It is important to note that our laboratory^11^ and Noh et al*.*^9^ have shown that the first dose of oral administration of ivabradine did not clearly have an anti-allodynic effect, but the anti-allodynic effects became evident after the second day of repeated oral administration in rodents with neuropathic pain. In contrast, ivabradine does not inhibit oxaliplatin-induced mechanical hyperalgesia in mice, although cold allodynia is abolished^13^. Thus, the animal studies have shown that ivabradine can alleviate some types of pain symptoms. These results suggest that repeated administration of ivabradine selectively and differentially attenuates neuropathic pain symptoms such as spontaneous pain, hyperalgesia and allodynia. However, the role of HCN channels in human pain states has rarely been investigated.

Topical application of capsaicin, which activates the transient receptor potential vanilloid 1 (TRPV1) channels expressed in primary sensory neurons, has been used as a human surrogate model of neuropathic pain with spontaneous pain, thermal hyperalgesia, secondary mechanical hyperalgesia and allodynia associated with neurogenic inflammation through peripheral and central mechanisms^14^. In the model, spontaneous pain and thermal hyperalgesia are mainly mediated by capsaicin-sensitive C-fibers^15,16^. Secondary mechanical hyperalgesia is considered to be mediated by capsaicin-insensitive A-fiber nociceptors, including Aδ-fiber high-threshold mechanoreceptors^17,18^. Dynamic mechanical allodynia in the secondary zone is mediated mainly by low-threshold mechanoreceptors (Aβ-fibers) ^19,20^. Our previous study showed that oral administration of ivabradine alleviates mechanical allodynia in rats with neuropathic pain via inhibition of the HCN current in large DRG neurons^11^, which are considered to be Aβ-fibers^21^.

We thus hypothesized that inhibition of HCN channels by repeated administration of ivabradine would affect pain perception in humans. Therefore, we investigated the effects of 2-day administration of ivabradine (total dose of 20 mg for 2 days) on capsaicin-induced pain perception in this randomized, double-blinded, placebo-controlled, crossover study in healthy volunteers.

## Results

### Participant characteristics

**Figure 1:**
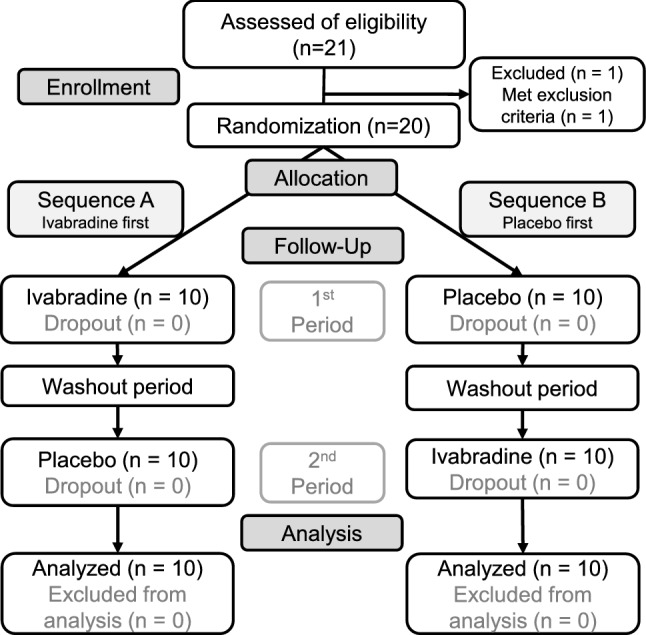
CONSORT diagram illustrating subject flow during the study.

**Figure 2:**
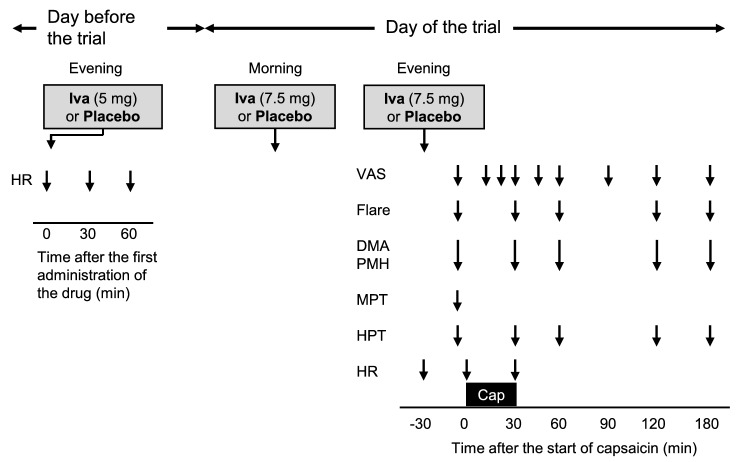
Timeline of the study period. Ivabradine (Iva) or a placebo was orally administered 3 times between the evening of the previous day and the evening of the trial day. Heart rate (HR) was measured before and 30 and 60 min after the first administration of the study drug (Iva or placebo) on the day before the capsaicin trial. HR was measured before and 30 and 60 min after the third administration of the study drug. After the third administration of the study drug, baseline assessment of spontaneous pain (VAS), flare size (Flare), area of dynamic mechanical allodynia using a foam brush (DMA), area of punctate mechanical hyperalgesia using a von Frey filament of 15 g (PMH), mechanical pain threshold (MPT) and heat-pain threshold (HPT) was done. Then a patch of filter paper (circle with a diameter of 2 cm) containing 0.5% capsaicin (100 μl) was placed for 30 min on the skin of the middle volar forearm of the dominant hand. The assessment was repeated up to 180 min after the start of capsaicin application. The bar labeled as “Cap” depicts the onset and duration of capsaicin application.

A total of 21 subjects were assessed for eligibility. One subject met the exclusion criteria because of regular use of analgesics. A total of 20 healthy subjects were randomly assigned to sequence A (ivabradine first, placebo second) or sequence B (placebo first, ivabradine second) and completed the study protocol consisting of two periods without missing data (Fig. 1). Table 1 shows characteristics of the subjects (Table 1). Data for 2 female and 18 male healthy subjects were finally analyzed. In this crossover trial, ivabradine or a placebo was administered three times in 2 days before capsaicin application (Fig. 2).

**Table 1:** Baseline characteristics.

Characteristic	Total (n = 20)	Sequence A (n = 10)	Sequence B (n = 10)	*p* value
Age (year)	28 ± 7	27 ± 5	29 ± 9	0.6932
Male sex, n (%)	18 (90)	9 (90)	9 (90)	> 0.99
Height (cm)	171 ± 6	170 ± 5	171 ± 6	0.6543
Weight (kg)	64 ± 8	63 ± 8	65 ± 8	0.6420
BMI (kg/m^2^)	22.1 ± 2.9	21.9 ± 2.5	22.3 ± 3.4	0.7850

A total of 20 healthy subjects were randomly assigned to sequence A (ivabradine first, placebo second) or sequence B (placebo first, ivabradine second). Data are expressed as means ± standard deviation unless otherwise indicated. There were no significant differences in baseline characteristics between the sequences. *BMI* body mass index.

### Carryover effect and period effect

There was no significant evidence of carryover effects in baseline heart rate, baseline heat-pain threshold, baseline mechanical pain threshold, and pain-related outcomes at the end of capsaicin application (30 min after the start of capsaicin application) (Supplementary Table S1), indicating that the reduction in heart rate caused by ivabradine and sensory changes after capsaicin application in the first period disappeared at the start of the second period. A statistically significant period effect was found only in the area of punctate mechanical hyperalgesia (*p* value for period effect = 0.0497). Therefore, pooled data from the first and second periods were used to investigate the effects of ivabradine on all of the outcomes except for the area of punctate mechanical hyperalgesia.

### Capsaicin-induced spontaneous pain

**Figure 3:**
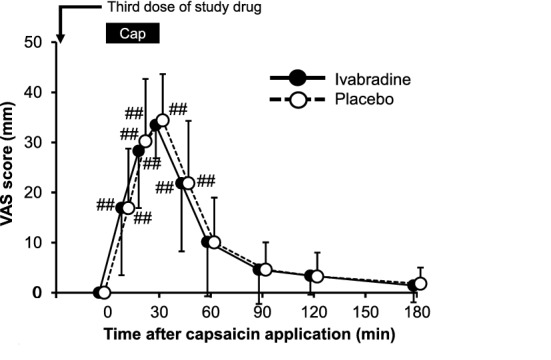
Effects of ivabradine and placebo on capsaicin-induced spontaneous pain assessed by visual analog scale (VAS) score. VAS scores in both groups gradually increased during the 30-min capsaicin application and gradually decreased after the end of capsaicin application. Data are expressed as means ± standard deviation, with n = 20 in each group. There were no significant differences in the VAS score between the groups throughout the study period. Statistical significance from baseline was indicated as ^#^*p* < 0.05 and ^##^*p* < 0.01. Statistical analysis was performed using two-way ANOVA with Scheffe’s multiple comparisons *post-hoc* test. The bar labeled as “Cap” at the top of the panel depicts the onset and duration of capsaicin application.

Capsaicin-induced spontaneous pain, which was defined as the primary outcome, gradually increased within the 30-min capsaicin application and gradually decreased after the end of capsaicin application (Fig. 3). There was no significant difference in the visual analog scale (VAS) for pain scores at 30 min between the ivabradine and placebo groups (33.5 ± 6.7 (mean ± standard deviation) vs. 34.4 ± 9.2 mm; F (1, 342) = 0.1035; 95% confidence interval (CI) for the difference, − 6.1 to 4.3 mm; *p* = 0.7479, n = 20 in each group) and at any other time during the study. Numerical data and results of statistical analysis are shown in Supplementary Table S2.

### Heat-pain threshold

**Figure 4:**
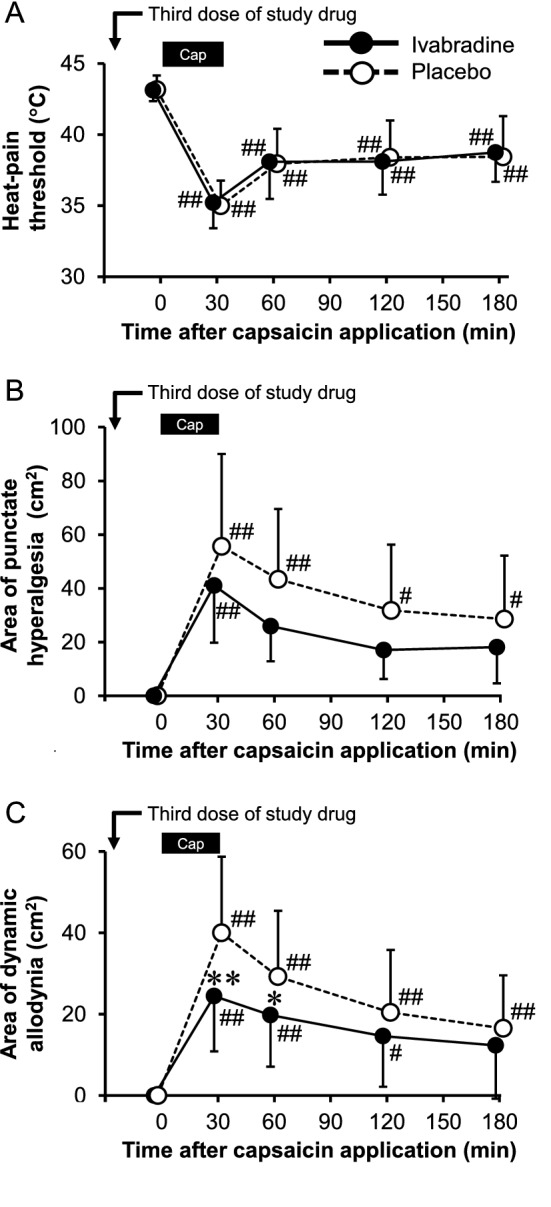
Effects of ivabradine and placebo on capsaicin-induced changes in heat sensitivity, and areas of punctate mechanical hyperalgesia and dynamic mechanical allodynia. Heat-pain threshold temperature was measured at the primary zone before and after capsaicin application and is presented in the panel (n = 20 in each group) (**A**). The area of punctate mechanical hyperalgesia was mapped using a von Frey filament of 15 g (n = 10 in each group) (**B**). The area of dynamic mechanical allodynia was mapped using a foam brush (n = 20 in each group) (**C**). Data are expressed as means ± standard deviation. Asterisks indicate statistically significant differences between the groups (*p < 0.05 and **p < 0.01). Statistical significance from baseline was indicated as #*p* < 0.05 and ##*p* < 0.01. Statistical analysis was performed using two-way ANOVA with Scheffe’s multiple comparisons *post-hoc* test. The bar labeled as “Cap” at the top of the panel depicts the onset and duration of capsaicin application.

Heat-pain threshold in both groups significantly decreased after capsaicin application compared to the baseline as shown in Fig. 4A. There were no significant differences at 30 min (F (1, 190) = 0.1017; 95% CI for the difference, − 1.0 to 1.4 °C; *p* = 0.7501, n = 20 in each group) and throughout the study period between the groups (Supplementary Table S2).

### Area of punctate mechanical hyperalgesia

There was a significant period effect in the area of punctate mechanical hyperalgesia by a von Frey filament of 15 g (Supplementary Table S1). Therefore, only data in the first period were used to investigate the effects of ivabradine and placebo on it. The area of secondary mechanical hyperalgesia at 30 min in the ivabradine group (41.1 ± 21.3 cm^2^) was slightly smaller than that in the placebo group (55.6 ± 34.4 cm^2^), but the difference was not statistically significant (F (1, 90) = 2.6792; 95% CI for the difference, − 41.4 to 12.3 cm^2^; *p* = 0.1052, n = 10 in each group) (Fig. 4B; Supplementary Table S3).

### Area of dynamic mechanical allodynia

The area of dynamic mechanical allodynia by brush stroking at 30 min in the ivabradine group (24.5 ± 13.6 cm^2^) was significantly smaller than that in the placebo group (40.0 ± 18.8 cm^2^) (F (1, 190) = 14.2152; 95% CI for the difference,  − 26.0 to − 5.0 cm^2^; *p* < 0.001, n = 20 in each group). The significant difference continued until 60 min after capsaicin application and then disappeared thereafter (Fig. 4C; Supplementary Table S2).

### Area of flare

**Figure 5:**
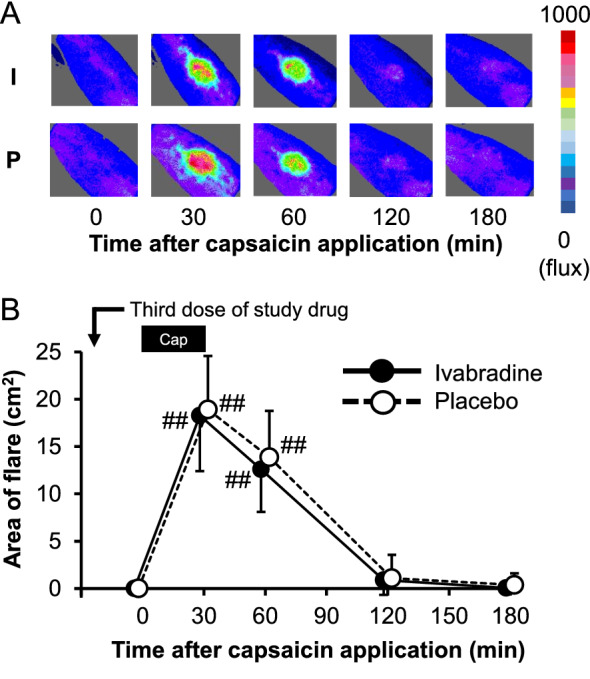
Effects of ivabradine and placebo on area of capsaicin-induced flare. Typical examples of laser-Doppler imaging pictures obtained from the same subject after ivabradine (I) and placebo (P) were presented in the panel (**A**). The increase in flux is indicated by a color code at the right of the panel. Panel (**B**) shows the time course of changes in flare size in both groups. Data are expressed as means ± standard deviation, with n = 20 in each group. There were no significant differences in flare size between the groups throughout the study period. Statistical significance from baseline was indicated as ^#^*p* < 0.05 and ^##^*p* < 0.01. Statistical analysis was performed using two-way ANOVA with Scheffe’s multiple comparisons *post-hoc* test. The bar labeled as “Cap” at the top of the panel depicts the onset and duration of capsaicin application.

Cutaneous flare response beyond the capsaicin application site was evident at 30 and 60 min as shown in Fig. 5A,B. There were no significant differences in flare size at 30 min between the groups (F (1, 190) = 0.3324; 95% CI for the difference, − 4.3 to 3.1 cm^2^; *p* = 0.5650, n = 20 in each group) (Supplementary Table S2).

### Changes in heart rate

There were no significant differences in heart rate before administration of the first study drug between the ivabradine group (68.5 ± 8.0 beat per minute (bpm)) and placebo group (67.7 ± 9.2 bpm) (95% CI for the difference, − 4.7 to 6.4 bpm; *p* = 0.7395, n = 20 in each group). After administration of ivabradine or placebo three times in 2 days, there were significant differences in mean heart rate between the ivabradine group (58.2 ± 4.7 bpm) and placebo group (65.3 ± 8.2 bpm) (95% CI for the difference, − 11.4 to − 2.9 bpm; *p* = 0.0043, n = 20 in each group) (see Supplementary Table S4).

### Safety

None of the subjects in the present study reported any adverse effects such as symptomatic bradycardia, visual side effects throughout the study and capsaicin-induced sensory changes that persisted beyond the next day.

## Discussion

The key feature of this study was that the effects of multiple doses of oral administration of ivabradine (a total dose of 20 mg in 2 days) on capsaicin-induced pain symptoms were investigated. Our results showed that there were no effects of ivabradine on capsaicin-induced spontaneous pain, which was defined as the primary outcome, reduction of heat-pain threshold at the site of capsaicin application, area of secondary punctate mechanical hyperalgesia, and area of flare. Only the area of secondary dynamic mechanical allodynia was significantly different between the ivabradine and placebo groups. In the present study, the effects of ivabradine on capsaicin-induced various sensory changes were evaluated under the condition in which repeated administration of ivabradine significantly reduced resting heart rate. It has been reported that ivabradine acts as an analgesic with a potency similar to its action as a bradycardic agent in rodents^10^. Orally administered ivabradine does not cross the blood–brain barrier^10,22^. In general, bloodborne substances have good access to the DRG^23^. Therefore, the roles of HCN channels primarily in DRG neurons in the capsaicin-induced pain state were investigated in this study.

Two-day administration of ivabradine was not effective for capsaicin-induced spontaneous pain, primary thermal hyperalgesia, and expansion of the area of punctate mechanical hyperalgesia. These findings are consistent with the results of a previous study by Lee et al.^24^ They reported that single oral administration of 15 mg of ivabradine, which was given 60 min before capsaicin application (0.5% cream) in healthy subjects, did not affect spontaneous pain, heat-pain threshold, or the area of punctate mechanical hyperalgesia induced by a 26-g von Frey filament^24^. Topical application of capsaicin to the skin evokes a short-lasting pain and thermal hyperalgesia at the application site, which are thought to be mostly mediated by TRPV1 channels in epidermal C-fibers^15,16^. Vasodilation beyond the site of capsaicin application is thought to be mainly mediated by calcitonin gene-related peptide, released from capsaicin-sensitive C-fiber terminals^17^. Importantly, the effect of ivabradine on capsaicin-induced increase in blood flow was quantified for the first time in our study using laser doppler flowmetry as shown Fig. 5. Our results obtained from various methods strongly suggest that HCN channels in DRG neurons have little involvement in the increased activity of capsaicin-sensitive fibers.

Central sensitization, induced by activation of TRPV1 on fine primary afferent nerve fibers, causes secondary mechanical hyperalgesia and allodynia beyond the area of capsaicin application^19^. Secondary hyperalgesia to punctate mechanical stimuli is thought to be mediated by capsaicin-insensitive A-fiber nociceptors, including Aδ-fiber high-threshold mechanoreceptors^17,18^. In addition to central sensitization, peripheral sensitization in an altered resting chemical environment in the skin in response to capsaicin application may also contribute to secondary mechanical hyperalgesia to some extent^25^. In the present study, ivabradine did not cause a significant reduction in the area of punctate mechanical hyperalgesia compared with that in placebo group, indicating that HCN channels in DRG neurons are not greatly involved in nociceptive signal transmission from the periphery to the spinal cord in the capsaicin pain model.

In the present study, only the areas of dynamic mechanical allodynia were different between the ivabradine and placebo groups, a finding that is not consistent with the results of a previous study in which the effects of a single dose of ivabradine were investigated^24^. Analgesic effects of some clinically available drugs for neuropathic pain such as pregabalin^26^ and amitriptyline^27^ become evident in patients after the second day of oral administration. Similarly, cumulative effects of oral ivabradine on mechanical allodynia (maximal effects being observed after 2–4 days) were reported in animal models with neuropathic pain^9,11^, indicating the possibility that repeated administration of ivabradine can produce stable concentrations of ivabradine and that suppression of HCN channels for a certain period of time is required to achieve an anti-allodynic effect. Non-noxious mechanical information is conveyed to the spinal dorsal horn mainly via low-threshold mechanoreceptors (Aβ-fibers) ^19,20^ but is pathologically converted to pain in the setting of neuropathic pain. A recent study showed that decreased function of inhibitory interneurons in the spinal dorsal horn of rodents with peripheral nerve injury is involved in conversion from non-painful to painful sensation^28^. The results of the present study are in line with the results of our previous study showing that oral administration of ivabradine alleviated mechanical allodynia in rats with neuropathic pain via inhibition of the HCN current in Aβ DRG neurons^11^. It is presumed that reduction of the area of dynamic mechanical allodynia observed in the present study is due to the inhibition of HCN channels in low-threshold mechanoreceptor neurons in the DRG.

In the present study, 2-day administration of ivabradine reduced the area of secondary dynamic mechanical allodynia but did not affect other symptoms in the capsaicin pain model. Our study did not reveal the mechanisms by which ivabradine reduces areas of secondary dynamic mechanical allodynia. As described in the introduction section, the analgesic effect of ivabradine depends on pain models and pain-inducing stimuli in animal studies. At present, it is not clear why analgesic effects of ivabradine vary depending on the pain symptoms. Differences in the distribution of HCN channels between nerve fibers may be one of the reasons for the different effects of ivabradine depending on types of neuropathic pain models and types of stimuli that evoke pain. Further studies with different designs would be necessary to investigate the mechanisms in detail.

It has been shown that 20–55% of patients with different causes of neuropathic pain had brush-evoked allodynia, although the prevalence of brush-evoked pain in neuropathic pain depends on the underlying condition^29,30^. Our results suggest that HCN channels may be a potential therapeutic target for the alleviation of mechanical allodynia. It seems to be difficult to further increase the dose of ivabradine because it blocks all HCN channels including HCN4 channels^5^, leading to reduction of heart rate. HCN1 and HCN2 are considered to be important in generating a hyperpolarization-activated inward-rectifying current in sensory neurons^2^. HCN1 is expressed in non-nociceptive DRG neurons with large myelinate (Aβ) axons. In contrast, HCN2 is expressed in nociceptive DRG neurons with small myelinated or unmyelinated axons^1^. The development of new highly selective blockers for HCN1 or HCN2 channels over HCN4 channels is required for the purpose of further investigating the involvement of HCN channels in mechanical allodynia.

There were some limitations in this study. The first is that we could not analyze the pooled data from the first and second periods to determine the effects of ivabradine on the area of punctate mechanical hyperalgesia because a significant period effect was found. Familiarization to punctate stimuli in conditions of its repetitions might have caused the significant period effect. Therefore, only data obtained in the first period were analyzed^31^. The second is that ivabradine was administered before capsaicin application in the present study. In other words, our study only showed the preventive effects of ivabradine on capsaicin-induced pain symptoms. It should be noted that it is not clear from the results of the present study whether ivabradine alleviates ongoing pain symptoms. Thirdly, we did not evaluate all of the sensory changes caused by capsaicin application. For example, the mechanical pain threshold was only measured before capsaicin application to investigate the carryover effect and period effect (Supplementary Table S1). Therefore, it is not clear how ivabradine affects capsaicin-induced changes in the mechanical pain threshold. Further studies are needed to delineate the effects of ivabradine on the sensory nervous system.

## Conclusions

Two-day oral administration of ivabradine, a nonselective HCN channel blocker, did not alleviate capsaicin-induced spontaneous pain and did not affect the decreased heat-pain threshold in the primary zone, the area of flare, and the area of punctate mechanical hyperalgesia in healthy humans. On the other hand, ivabradine reduced the areas of dynamic mechanical allodynia. HCN channels may be differentially involved in the various pain signal transmission pathways.

## Methods

### Study design and participants

This randomized, double-blind, crossover study was approved by the Institutional Ethics Committee of Shinshu University School of Medicine, Matsumoto, Japan (document number: 3439). The study was registered in the University Hospital Medical Information Network (UMIN) in Japan, number UMIN000023022 on 05/07/2016 (https://center6.umin.ac.jp/cgi-open-bin/ctr_e/ctr_view.cgi?recptno=R000026532). The study was carried out in Shinshu University Hospital from January 2017 to May 2018. Subjects were recruited by advertisement at our university hospital. Written informed consent was obtained from all participants. The study was conducted in accordance with the principles outlined in the Declaration of Helsinki.

### Inclusion and exclusion criteria

The inclusion criterion was adults (20 years of age or older) with generally good health according to their medical histories and without diseases that were being treated. Exclusion criteria were: (1) current ongoing pain, (2) bradycardia (heart rate less than 55 bpm at baseline), (3) cardiac arrhythmias, (4) neurologic deficits, (5) psychotic and depressive disorders based on medical histories, (6) current or regular intake of any drugs that might affect pain or nociception, and (7) pregnant or lactating women.

### Randomization and blinding

Subjects were randomly assigned to either sequence A (ivabradine first, then placebo) or sequence B (placebo first, then ivabradine) using a computer randomization (block size: 4) with an allocation ratio of 1:1 by an author who was not involved in data collection. All outcome assessors as well as the participants were blind to the study group. The drugs were wrapped in the same type of capsule by investigators who did not participate in the outcome evaluation.

### Ivabradine

At the beginning of this study, ivabradine was not clinically available in Japan. After getting an import permit (no. 36011), issued by Ministry of Health, Labour and Welfare, for use only in this study, we imported ivabradine 5 mg and 7.5 mg tablets (Procoralan®, Servier, Suisse), which were approved for clinical use in Europe.

### Topical application of capsaicin

The application site of capsaicin was marked midway between the elbow and the wrist of the dominant volar forearm. Topical application of capsaicin was performed according to a previously described method^32^. A solution of 0.5% (5 mg/ml) capsaicin (Sigma, Tokyo, Japan) in 50% ethanol and 50% distilled water was prepared and 100 μl of the solution was absorbed onto a round-shaped filter paper with a diameter of 2 cm. The filter paper containing capsaicin was applied to the predetermined site and then covered by a piece of transparent adhesive film dressing for 30 min to avoid evaporation. After that, the filter paper was removed from the forearm. The skin temperature at the capsaicin application site was measured before the application and was kept between 31 and 34 °C in all subjects in a quiet room with a temperature of 22–24 °C.

### Study protocol

In this crossover study, each subject participated in two periods separated by a washout period of more than 7 days. The elimination half-life of ivabradine in healthy humans is less than 2 h^33^ and it is considered that the washout period is at least five times of the half-life of the drug^34^. The study protocol in one period is shown in Fig. 2. Subjects were familiarized with the study protocol and the sensory tests described later. The therapeutic dose of ivabradine for chronic stable angina ranges between 2.5 and 7.5 mg administered twice daily^8^. The clinical maximum dose of ivabradine is 15 mg/day (7.5 mg twice daily)^8^. On the evening before the trial day of capsaicin application, the first dose of study drug (ivabradine 5 mg or placebo) was orally administered in a room of the hospital. Resting heart rate was measured by using a pulse oximeter before and 30 and 60 min after the first dose while evaluating the presence or absence of adverse events. At 7:00 in the morning of the trial day, the subjects were given the second dose of study drug (ivabradine 7.5 mg or placebo). At 17:00 in the evening, heart rate was measured again before the subjects received the third dose of study drug (ivabradine 7.5 mg or placebo) in the room of the hospital. The time to peak plasma concentration of oral ivabradine is approximately 0.7 h^35,36^. Therefore, capsaicin application was started at 30 min after the third oral administration of ivabradine. Capsaicin was topically applied for 30 min.

### Study outcomes

The primary outcome measure was capsaicin-induced spontaneous pain at 30 min after capsaicin application. The secondary outcome measures included heat-pain threshold, area of mechanical hyperalgesia/allodynia and area of the flare response. Exploratory outcomes included changes in heart rate.

### Spontaneous pain assessment

Subjects rated their perception of spontaneous pain using a VAS consisting of a 100-mm line, with 0 representing “no pain” and 100 representing “pain as worst imaginable”.

### Heat-pain threshold

Heat-pain threshold at the capsaicin application site (primary zone) was measured by using a thermal stimulator with a round-shaped thermode with a diameter of 5 mm (Thermal Stimulator UDH-300, Unique Medical, Co, Tokyo, Japan). Baseline temperature was set at 32 °C and ramp rate was fixed at 1 °C/s with a cut-off temperature of 50 °C. The subjects were instructed to release a button (which returns the temperature to 32 °C) as soon as they perceived painful sensation.

### Mechanical pain threshold

Mechanical pain threshold was assessed by using the up-down method as previously described^37,38^. Calibrated von Frey fibers (Stoelting Co., Wood Dale, IL USA) were applied to the scheduled capsaicin application site in the volar forearm until the fiber bent slightly for 2 s. The testing was initiated using a von Frey filament of 8 g. This up-down procedure was applied 6 times after the first positive response. Mechanical pain threshold was calculated using a coefficient based on the response pattern^37,38^.

### Dynamic mechanical allodynia and punctate mechanical hyperalgesia

Eight radial grid lines with dots at 1-cm intervals were drawn from the capsaicin application site on the skin. The area of dynamic mechanical allodynia was mapped by gently stroking a 1-in. foam brush^39^. The brush stimulation was moved at a rate of approximate 1 cm/s^40^ in steps of 0.5 cm along the 8 radial lines from the nonpainful area toward the application site until the subject reported pain or tenderness and the distance from the center was recorded. The procedures were repeated for all of the grid lines. The area of punctate mechanical hyperalgesia was assessed by a 15-g von Frey filament^39^. The punctate probe was moved in steps of 0.5 cm along the 8 radial lines from the nonpainful or slightly painful area toward the application site until the subject reported a distinct increase in pain (hyperalgesia) compared to the previous stimulation or when there was a change in sensation from a non-painful to a painful sensation. The area of an octagon with 8 vertices was calculated (see Supplementary Figure S1).

### Area of a flare response

Superficial skin blood flow in and around the capsaicin application site was assessed using a laser-Doppler imager (moorFLPI-2, moor instruments, Axminster, UK). Blood flux at each pixel was analyzed offline by using MoorFLPI review (version 4.0) software. The area of flare was defined as the skin area with a blood flux exceeding the mean flux plus twofold standard deviation assessed at baseline^41^.

### Safety assessment

A medical history was obtained and an electrocardiogram test was performed at a screening session. Subjects was closely monitored for 60 min after the first administration of the study drug. On the day after capsaicin application, we asked the subjects by phone about their general condition and about pain and discomfort around the capsaicin application site.

### Sample size

A previous study performed by Zheng et al.^32^, in which a similar method for capsaicin application was used, showed that the mean VAS score and its standard deviation at 30 min after capsaicin application were approximately 45 and 15 mm, respectively. It has been reported that pain reduction of 30% represents a clinically meaningful effect^42^. To detect the difference between the groups, sample size was calculated by using the G*Power program (version 3.1). With a type I error of 0.05 and power of 0.8, the required sample size was 17. Considering a dropout rate of 15%, a sample size of 20 subjects was determined to be necessary of this study.

### Carryover effect and period effect

In order to check the possible carryover effects from one treatment period to the next, the sum of the scores measured in the two periods was compared between the two sequence groups by using the *t*-test. In addition, the period effect was tested by comparing the valuables in the first period over the second one^31^.

### Statistical analysis

After the Shapiro–Wilk test of normality, data were expressed as mean ± standard deviation or median [25%, 75% interquartile range], as appropriate. All of the data except for mechanical pain threshold data were compared by two-way ANOVA for repeated measures with Scheffe’s post hoc test. Data for mechanical pain threshold were analyzed by the Mann–Whitney U test. Percentages of male and female subjects were compared by a chi-square test. Subject characteristics were compared using the unpaired t-test. *p* < 0.05 was considered as statistically significant. All statistical tests were done with a two-tailed hypothesis and performed using Bell Curve for Excel software, version 3.21 (Social Survey Research Information Co., Ltd., Tokyo, Japan).

## Supplementary Information

Supplementary Information.

## Data Availability

The datasets generated during and analyzed during the current study are not publicly available but are available from the corresponding author on reasonable request.

## References

[1] Benarroch EE (2013). HCN channels: Function and clinical implications. Neurology.

[2] Finnerup NB, Kuner R, Jensen TS (2021). Neuropathic pain: From mechanisms to treatment. Physiol. Rev..

[3] Sartiani L, Mannaioni G, Masi A, Romanelli MN, Cerbai E (2017). The hyperpolarization-activated cyclic nucleotide-gated channels: From biophysics to pharmacology of a unique family of ion channels. Pharmacol. Rev..

[4] Tibbs GR, Posson DJ, Goldstein PA (2016). Voltage-gated ion channels in the PNS: Novel therapies for neuropathic pain?. Trends Pharmacol. Sci..

[5] Stieber J, Wieland K, Stöckl G, Ludwig A, Hofmann F (2006). Bradycardic and proarrhythmic properties of sinus node inhibitors. Mol. Pharmacol..

[6] Swedberg K, SHIFT Investigators (2010). Ivabradine and outcomes in chronic heart failure (SHIFT): A randomised placebo-controlled study. Lancet.

[7] Cao Y, Pang J, Zhou P (2016). HCN channel as therapeutic targets for heart failure and pain. Curr. Top. Med. Chem..

[8] Koruth JS, Lala A, Pinney S, Reddy VY, Dukkipati SR (2017). The clinical use of ivabradine. J. Am. Col. Cardiol..

[9] Noh S (2014). The heart-rate-reducing agent, ivabradine, reduces mechanical allodynia in a rodent model of neuropathic pain. Eur. J. Pain.

[10] Young GT, Emery EC, Mooney ER, Tsantoulas C, McNaughton PA (2014). Inflammatory and neuropathic pain are rapidly suppressed by peripheral block of hyperpolarisation-activated cyclic nucleotide-gated ion channels. Pain.

[11] Zhang H (2019). Prostanoid EP4 receptor-mediated augmentation of *I*
_h_ currents in Aβ dorsal root ganglion neurons underlies neuropathic pain. J. Pharmacol. Exp. Ther..

[12] Miyake S (2019). Locally injected ivabradine inhibits carrageenan-induced pain and inflammatory responses via hyperpolarization-activated cyclic nucleotide-gated (HCN) channels. PLoS One.

[13] Descouer J (2011). Oxaliplatin-induced cold hypersensitivity is due to remodelling of ion channel expression in nociceptors. EMBO. Mol. Med..

[14] Olesen AE, Andresen T, Staahl C, Drewes AM (2012). Human experimental pain models for assessing the therapeutic efficacy of analgesic drugs. Pharmacol. Rev..

[15] LaMotte RH, Shain CN, Simone DA, Tsai EF (1991). Neurogenic hyperalgesia: Psychophysical studies of underlying mechanisms. J. Neurophysiol..

[16] Van der Schueren BJ (2008). Calcitonin gene-related peptide 8–37 antagonizes capsaicin-induced vasodilation in the skin: Evaluation of a human in vivo pharmacodynamic model. J. Pharmacol. Exp. Ther..

[17] Ziegler EA, Magerl W, Meyer RA, Treede RD (1999). Secondary hyperalgesia to punctate mechanical stimuli. Central sensitization to A-fibre nociceptor input. Brain.

[18] Magerl W, Fuchs PN, Meyer RA, Treede RD (2001). Roles of capsaicin-insensitive nociceptors in cutaneous pain and secondary hyperalgesia. Brain.

[19] Treede RD, Cole JD (1993). Dissociated secondary hyperalgesia in a subject with a large-fibre sensory neuropathy. Pain.

[20] La JH, Chung JM (2017). Peripheral afferents and spinal inhibitory system in dynamic and static mechanical allodynia. Pain.

[21] Lawson SN, Fang X, Djouhri L (2019). Nociceptor subtypes and their incidence in rat lumbar dorsal root ganglia (DRGs): Focussing on C-polymodal nociceptors, Aβ-nociceptors, moderate pressure receptors and their receptive field depths. Curr. Opin. Physiol..

[22] Savelieva I, Camm AJ (2006). Novel If current inhibitor ivabradine: Safety considerations. Adv. Cardiol..

[23] Abram SE, Yi J, Fuchs A, Hogan QH (2006). Permeability of injured and intact peripheral nerves and dorsal root ganglia. Anesthesiology.

[24] Lee MC (2019). A randomised, double-blind, placebo-controlled crossover trial of the influence of the HCN channel blocker ivabradine in a healthy volunteer pain model: An enriched population trial. Pain.

[25] Serra J, Campero M, Bostock H, Ochoa J (2004). Two types of C nociceptors in human skin and their behavior in areas of capsaicin-induced secondary hyperalgesia. J. Neurophysiol..

[26] Sharma U, Griesing T, Emir B, Young JP (2010). Time to onset of neuropathic pain reduction: A retrospective analysis of data from nine controlled trials of pregabalin for painful diabetic peripheral neuropathy and postherpetic neuralgia. Am. J. Ther..

[27] Kautio AL, Haanpää M, Saarto T, Kalso E (2008). Amitriptyline in the treatment of chemotherapy-induced neuropathic symptoms. J. Pain Symptom Manage..

[28] Tashima R (2021). A subset of spinal dorsal horn interneurons crucial for gating touch-evoked pain-like behavior. Proc. Natl. Acad. Sci. USA.

[29] Attal N (2008). Neuropathic pain: Are there distinct subtypes depending on the aetiology or anatomical lesion?. Pain.

[30] Maier C (2010). Quantitative sensory testing in the German Research Network on Neuropathic Pain (DFNS): Somatosensory abnormalities in 1236 patients with different neuropathic pain syndromes. Pain.

[31] Wellek S, Blettner M (2012). On the proper use of the crossover design in clinical trials: Part 18 of a series on evaluation of scientific publications. Dtsch Ärztebl. Int..

[32] Zheng Z, Gibson SJ, Helme RD, McMeeken JM (2009). The effect of local anaesthetic on age-related capsaicin-induced mechanical hyperalgesia—a randomised, controlled study. Pain.

[33] Vlase L (2011). Pharmacokinetic interaction between ivabradine and carbamazepine in healthy volunteers. J. Clin. Pharm. Ther..

[34] Dhariwal K, Jackson A (2003). Effect of length of sampling schedule and washout interval on magnitude of drug carryover from period 1 to period 2 in two-period, two-treatment bioequivalence studies and its attendant effects on determination of bioequivalence. Biopharm. Drug Dispos..

[35] Choi HY (2013). Evaluation of pharmacokinetic and pharmacodynamic profiles and tolerability after single (2.5, 5, or 10 mg) and repeated (2.5, 5, or 10 mg bid for 4.5 days) oral administration of ivabradine in healthy male Korean volunteers. Clin. Ther..

[36] Jiang J, Tian L, Huang Y, Li Y, Xu L (2013). Pharmacokinetic and safety profile of ivabradine in healthy Chinese men: A phase I, randomized, open-label, increasing single- and multiple-dose study. Clin. Ther..

[37] Chaplan SR, Bach FW, Pogrel JW, Chung JM, Yaksh TL (1994). Quantitative assessment of tactile allodynia in the rat paw. J. Neurosci. Methods.

[38] Gonzalez-Cano R, Boivin B, Bullock D, Cornelissen L, Andrews N, Costigan M (2018). Up-down reader: An open source program for efficiently processing 50% von frey thresholds. Front. Pharmacol..

[39] Wong W, Wallace MS (2014). Determination of the effective dose of pregabalin on human experimental pain using the sequential up-down method. J. Pain.

[40] Gottrup H (2004). Chronic oral gabapentin reduces elements of central sensitization in human experimental hyperalgesia. Anesthesiology.

[41] Krämer HH, Schmelz M, Birklein F, Bickel A (2004). Electrically stimulated axon reflexes are diminished in diabetic small fiber neuropathies. Diabetes.

[42] Farrar JT, Young JP, LaMoreaux L, Werth JL, Poole MR (2001). Clinical importance of changes in chronic pain intensity measured on an 11-point numerical pain rating scale. Pain.

