# Supplementary material for: A randomized, double-blinded, placebo-controlled, crossover study of the HCN channel blocker ivabradine in a capsaicin-induced pain model in healthy volunteers
Source: Sci Rep. 2022 Oct 14;12:17246. doi: 10.1038/s41598-022-22309-7 (PMC9568658; doi:10.1038/s41598-022-22309-7)
Supplement: Supplementary file 1 — Supplementary Information. [file 41598_2022_22309_MOESM1_ESM.docx]

**Supplementary information file**

**A randomized, double-blinded, placebo-controlled, crossover study of the HCN channel blocker ivabradine in a capsaicin-induced pain model in healthy volunteers**

Satoshi Tanaka^1 *^, Takashi Ishida^1^, Kumiko Ishida^1^, Satoshi Fuseya^1^, Mariko Ito^1^, Akiyuki Sakamoto^1^, and Mikito Kawamata^1^

**^1^** Department of Anesthesiology and Resuscitology, Shinshu University School of Medicine, Matsumoto, Nagano, Japan.

^*^Corresponding Author, Satoshi Tanaka: s_tanaka@shinshu-u.ac.jp

This supplementary file includes the following data:

1. Supplementary **Table S1.** Carryover effect and period effect
2. Supplementary **Table S2.** Effects of ivabradine and placebo in combined data from the first and second periods
3. Supplementary **Table S3.** Effects of ivabradine and placebo on the area of punctate mechanical hyperalgesia in the first period.
4. Supplementary **Table S4.** Changes in heart rate during the study period
5. Supplementary **Figure S1.** Schematic representation of the areas of secondary mechanical hyperalgesia and allodynia on the volar forearm after the capsaicin application

**Supplementary Table S1.** Carryover effect and period effect

|  | **Sequence A (n = 10)** | | **Sequence B (n = 10)** | | ***p* value for Carryover effect** | ***p* value for Period effect** |
| --- | --- | --- | --- | --- | --- | --- |
| **Variable** | **1st period**  **(Ivabradine)** | **2nd period**  **(Placebo)** | **1st period**  **(Placebo)** | **2nd period**  **(Ivabradine)** |  |  |
| At baseline  HR (bpm) before first study drug  HPT (°C) before capsaicin  MPT (g) before capsaicin | 68.8 ± 7.5  43.2 ± 0.8  11.1 [8.4, 15.6] | 68.5 ± 7.2  43.3 ± 1.0  12.5 [8.5, 17.9] | 66.9 ± 11.1  43.1 ± 1.1  9.1 [7.2, 11.2] | 68.3 ± 8.9  43.1 ± 0.8  9.8 [7.8, 10.9] | 0.7582  0.6863  0.1677 | 0.7896  0.8314  0.1366 |
| At 30 min after capsaicin  VAS (mm)  Changes in HR (bpm)  Changes in HPT (°C)  Area of flare (cm^2^)  Area of hyperalgesia (cm^2^)  Area of allodynia (cm^2^) | 35.6 ± 6.4  -10.4 ± 8.2  -8.5 ± 1.4  18.1 ± 6.3  41.1 ± 21.3  24.1 ± 11.6 | 34.6 ± 8.8  -1.9 ± 6.8  -8.4 ± 1.9  18.7 ± 5.9  46.0 ± 21.6  37.1 ± 17.0 | 34.2 ± 10.1  -2.8 ± 6.6  -7.9 ± 1.3  19.1 ± 5.7  55.6 ± 34.4  42.9 ± 20.8 | 31.4 ± 6.6  -10.4 ± 6.8  -7.3 ± 2.3  18.5 ± 5.8  36.5 ± 25.9  24.8 ± 16.0 | 0.4498  0.8547  0.2416  0.8611  0.8245  0.6560 | 0.3734  0.8329  0.3652  0.9850  **0.0497***  0.1877 |

Data are expressed as means ± standard deviation or medians [25%, 75% interquartile range]. Changes in HR are the differences in heart rate before the first study drug (ivabradine or placebo) and 30 min after the start of capsaicin application. Changes in HPT are the differences in heat-pain threshold before capsaicin application and 30 min after the start of capsaicin application. There were no significant carryover effects in all variables. A significant period effect was found in only the area of punctate mechanical hyperalgesia, as shown in bold with an asterisk (*). Abbreviations: HR, heart rate; bpm, beats per minute; HPT, heat-pain threshold; MPT, mechanical pain threshold; VAS, visual analogue scale for pain.

**Supplementary Table S2.** Effects of ivabradine and placebo in combined data from the first and second periods

| **Variable** | **Time after capsaicin application** | **Ivabradine**  **(n = 20)** | | **Placebo**  **(n = 20)** | **Statistic F** | ***p* value** | **95% CI for difference** |
| --- | --- | --- | --- | --- | --- | --- | --- |
| VAS (mm) | Baseline  10 min  20 min  30 min  45 min  60 min  90 min  120 min  180 min | 0 ± 0  17.0 ± 13.5  28.3 ± 11.4  33.5 ± 6.7  21.9 ± 13.6  10.2 ± 10.9  4.6 ± 6.8  3.4 ± 3.8  1.5 ± 3.3 | 0 ± 0  16.9 ± 11.9  30.3 ± 12.4  34.4 ± 9.2  21.9 ± 12.4  10.1 ± 8.9  4.6 ± 5.5  3.3 ± 4.8  1.8 ± 3.2 | | -  F (1, 342) = 0.0003  F (1, 342) = 0.4857  F (1, 342) = 0.1035  F (1, 342) = 0.0000  F (1, 342) = 0.0013  F (1, 342) = 0.0000  F (1, 342) = 0.0029  F (1, 342) = 0.0156 | -  0.9858  0.4863  0.7479  >0.99  0.9715  >0.99  0.9573  0.9005 | -  -8.1 to 8.3  -9.6 to 5.6  -6.1 to 4.3  -8.4 to 8.4  -6.3 to 6.5  -4.0 to 4.0  -2.7 to 2.9  -2.4 to 1.8 |
| HPT (°C) | Baseline  30 min  60 min  120 min  180 min | 43.1 ± 0.8  35.2 ± 1.8  38.1 ± 2.6  38.1 ± 2.3  38.7 ± 2.1 | 43.2 ± 1.0  35.0 ± 1.8  38.0 ± 2.5  38.4 ± 2.6  38.4 ± 2.9 | | F (1, 190) = 0.0009  F (1, 190) = 0.1017  F (1, 190) = 0.0372  F (1, 190) = 0.1787  F (1, 190) = 0.1981 | 0.9764  0.7501  0.8473  0.6729  0.6568 | -0.7 to 0.5  -1.0 to 1.4  -1.5 to 1.7  -1.9 to 1.3  -1.3 to 1.9 |
| Area of DMA (cm^2^) | Baseline  30 min  60 min  120 min  180 min | 0 ± 0  24.5 ± 13.6  19.8 ± 12.7  14.6 ± 12.5  12.3 ± 13.1 | 0 ± 0  40.0 ± 18.8  29.2 ± 16.2  20.4 ± 15.4  16.5 ± 13.0 | | -  F (1, 190) = 14.2152  F (1, 190) = 5.2804  F (1, 190) = 1.9934  F (1, 190) = 1.0407 | -  **<0.001***  **0.0227***  0.1596  0.3090 | -  -26.0 to -5.0  -18.8 to -0.1  -14.8 to 3.2  -12.6 to 4.2 |
| Area of flare (cm^2^) | Baseline  30 min  60 min  120 min  180 min | 0 ± 0  18.3 ± 5.9  12.6 ± 4.5  0.9 ± 1.6  0.1 ± 0.2 | 0 ± 0  18.9 ± 5.7  13.9 ± 4.9  1.1 ± 2.5  0.4 ± 1.2 | | -  F (1, 190) = 0.3324  F (1, 190) = 1.3347  F (1, 190) = 0.0359  F (1, 190) = 0.0765 | -  0.5650  0.2494  0.8499  0.7823 | -  -4.3 to 3.1  -4.3 to 1.8  -1.5 to 1.1  -0.9 to 0.3 |

Data are expressed as means ± standard deviation. All significant results at *p* < 0.05 are shown in bold with asterisks (*).

Abbreviations: VAS, visual analogue scale for pain; HPT, heat-pain threshold; DMA, dynamic mechanical allodynia; CI, confidence interval.

**Supplementary Table S3**. Effects of ivabradine and placebo on the area of punctate mechanical hyperalgesia in the first period

| **Variable** | **Time after capsaicin application** | **Sequence A**  **Ivabradine**  **(1st period)**  **(n = 10)** | **Sequence B**  **Placebo**  **(1st period)**  **(n = 10)** | **Statistic F** | ***p* value** | **95% CI for difference** |
| --- | --- | --- | --- | --- | --- | --- |
| Area　of PMH (cm^2^) | Baseline  30 min  60 min  120 min   1. min | 0 ± 0  41.1 ± 21.3  26.0 ± 13.1  17.1 ± 10.8  18.2 ± 13.5 | 0 ± 0  55.6 ± 34.4  43.4 ± 26.2  31.8 ± 24.5  28.6 ± 23.6 | -  F (1, 90) = 2.6792  F (1, 90) = 3.8314  F (1, 90) = 2.7507  F (1, 90) = 1.3877 | -  0.1052  0.0534  0.1007  0.2419 | -  -41.4 to 12.3  -36.8 to 2.0  -32.5 to 3.0  -28.5 to 7.6 |

Data are expressed as means ± standard deviation. There were no significant differences in the area of PMH between the ivabradine and placebo groups throughout the study period. Abbreviations: PMH, punctate mechanical hyperalgesia; CI, confidence interval.

**Supplementary Table S4.** Changes in heart rate during the study period

| **Variable** | **Measurement timing** | **Ivabradine**  **(n = 20)** | **Placebo**  **(n = 20)** | ***p* value** | **95% CI for difference** |
| --- | --- | --- | --- | --- | --- |
| HR (bpm) | Day before capsaicin application  before the first drug  30 min after the first drug  60 min after the first drug  Day of capsaicin application  30 min before capsaicin  At the start of capsaicin  30 min after capsaicin | 68.5 ± 8.0  67.1 ± 8.7  64.9 ± 7.5  62.5 ± 4.9  59.4 ± 5.5  58.2 ± 4.7 | 67.7 ± 9.2  66.5 ± 9.5  66.9 ± 9.6  66.5 ± 7.3  65.6 ± 8.8  65.3 ± 8.2 | 0.7395  0.8245  0.4204  0.1123  **0.0123***  **0.0043*** | -4.7 to 6.4  -5.3 to 6.4  -7.5 to 3.5  -8.0 to 0.1  -10.9 to -1.6  -11.4 to -2.9 |

Data are expressed as means ± standard deviation. All significant results at *p* < 0.05 between the groups are shown in bold with asterisks (*). Abbreviations: HR, heart rate; bpm, beats per minute; CI, confidence interval.


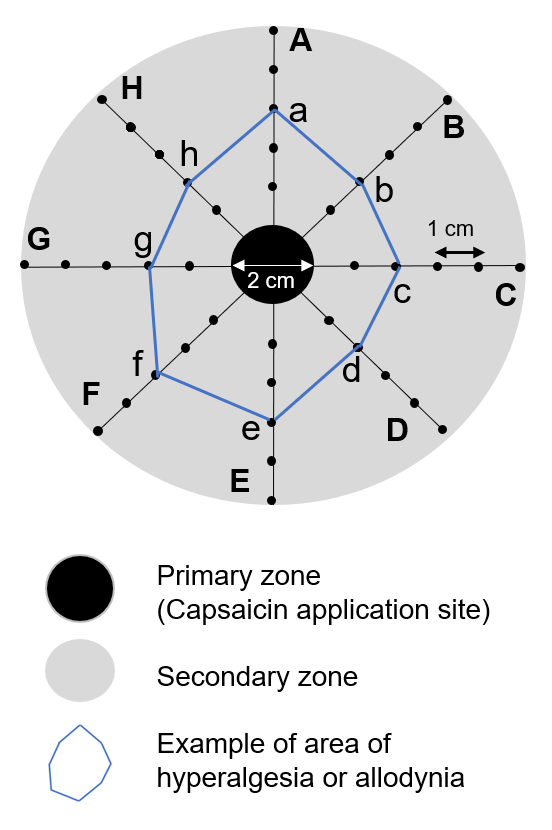


**Supplementary Figure S1.** Schematic representation of the areas of secondary mechanical hyperalgesia and allodynia on the volar forearm after capsaicin application. Eight radial grid lines with dots at 1-cm intervals were drawn on the skin of the middle volar forearm of the dominant hand. A patch of filter paper (circle with a diameter of 2 cm) containing 0.5% capsaicin (100 μl) was placed for 30 min (primary zone). A 15 g von Frey filament (for punctate mechanical hyperalgesia) or a foam brush (for dynamic mechanical allodynia) was used to apply stimuli along each grid line from the periphery towards the center of the grid. The point was recorded when the subject reported a distinct increase in pain (hyperalgesia) compared to the previous stimulation or when there was a change in sensation from a non-painful to a painful sensation (allodynia). The procedures were repeated for all of the grid lines (from A to H). Area of the octagon with 8 vertices (a, b, c, d, e, f, g, and h) was calculated as follows: Area = sin 45 degrees × (a × b + b × c + c × d + d × e + e × f + f × g + g × h + h × a)/2. In the example in this figure, sin (45) × (4 × 3 + 3 × 3 + 3 × 3 + 3 × 4 + 4 × 4 + 4 × 3 + 3 × 3 + 3 × 4)/2 = 32. 1 cm^2^.
